# Adversities Mediate Social Determinants of Youth Tobacco Use Initiation

**DOI:** 10.31586/jbls.2024.1039

**Published:** 2024-08-29

**Authors:** Shervin Assari, Payam Sheikhattari, Hossein Zare

**Affiliations:** 1 Department of Internal Medicine, Charles R. Drew University of Medicine and Science, Los Angeles, CA, United States; 2 Department of Family Medicine, Charles R. Drew University of Medicine and Science, Los Angeles, CA, United States; 3 Department of Urban Public Health, Charles R. Drew University of Medicine and Science, Los Angeles, CA, United States; 4 Marginalization-Related Diminished Returns (MDRs) Center, Los Angeles, CA, United States; 5 Center for Urban Health Disparities Research and Innovation, Morgan State University, Baltimore, MD, USA; 6 The Prevention Sciences Research Center, School of Community Health and Policy, Morgan State University, Baltimore, MD, USA; 7 Department of Behavioral Health Science, School of Community Health and Policy, Morgan State University, Baltimore, MD, USA; 8 Department of Health Policy and Management, Johns Hopkins Bloomberg School of Public Health, Baltimore, MD, United States; 9 School of Business, University of Maryland Global Campus (UMGC), College Park, MD, United States

**Keywords:** Social Determinants of Health, Youth Tobacco Use, Adversities, Mediation, Parental Education, Household Income, Neighborhood Income, Family Structure, Perceived Discrimination, Life Trauma, Financial Strain

## Abstract

**Background:**

Social determinants of health (SDOH) significantly influence health behaviors, including tobacco use among youth. Adversities such as perceived discrimination, perceived neighborhood stress, life trauma, and financial strain are stressors that may mediate the relationship between various SDOH and youth tobacco use. This study aims to investigate whether multidimensional adversities mediate the effects of SDOH on tobacco use among youth.

**Methods:**

Data from the Adolescent Brain Cognitive Development (ABCD) study were used to test our hypotheses. The sample included a diverse cohort of youth aged 9-10 years old followed until they were 15-16 years old. We examined the effects of baseline parental education, household income, neighborhood income, and family structure on subsequent youth tobacco use. Structural equation models were used to test if adversities (perceived discrimination, life trauma, financial strain) operate as potential mediators.

**Results:**

All ABCD participants were eligible for our analysis, regardless of race, ethnicity, or SDOHs (n = 11,878). The findings indicated that the effects of parental education, household income, neighborhood income, and family structure on youth tobacco use were partially mediated by adversities. Higher levels of parental education and household income were associated with lower tobacco use, and this relationship was weakened when accounting for adversities. Similarly, stable family structures and higher neighborhood income were linked to reduced tobacco use, with adversities playing a mediating role.

**Conclusions:**

Multidimensional adversities partially mediate the relationship between SDOH at baseline and subsequent youth tobacco use. Interventions aimed at reducing youth tobacco use should address both the social determinants and multiple adversities experienced by adolescents. Policies to improve the educational and economic situations of families, enhance neighborhood environments, and support stable family structures all reduce youth tobacco use, with lower exposure to adversities explaining this effect.

## 1. Introduction

Social determinants of health (SDOH) are the non-medical factors that influence health outcomes [1-3]. These include conditions in which people are born, grow, live, work, and age, as well as the broader set of forces and systems shaping the conditions of daily life [4-6]. These forces and systems include economic policies and systems, development agendas, social norms, social policies, and political systems [4, 7, 8]. Understanding the impact of SDOH is critical for developing effective interventions to improve health outcomes and reduce health disparities.

Youth tobacco use is a significant public health concern with long-term health consequences [9-11]. Various SDOH, such as parental education, household income, neighborhood socioeconomic status, and family structure, play crucial roles in shaping health behaviors, including tobacco use among youth [12-14]. Higher levels of parental education and household income, as well as more stable family structures, are generally associated with lower rates of youth tobacco use [15-19]. Conversely, lower neighborhood socioeconomic status [20-24] and less stable family structures [25-27] may increase the risk of tobacco use among young people. Understanding these associations helps in identifying at-risk groups and developing targeted prevention strategies.

Adverse experiences, including perceived discrimination, life trauma, and financial strain, are significant stressors that can influence health behaviors, including tobacco use [28-34]. These adversities can lead to increased stress and psychological distress, which in turn may promote tobacco use as a coping mechanism [35-37]. Adolescents facing multiple adversities are particularly vulnerable to initiating tobacco use, which can have lasting impacts on their health and well-being. Investigating the role of adversities in tobacco use among youth is essential for developing comprehensive prevention and intervention strategies [38-41].

## 2. Aim and Hypothesis

The aim of this study is to investigate whether adversities mediate the effects of social determinants of health on tobacco use among youth. We hypothesize that the associations between SDOH (parental education, household income, neighborhood income, and family structure) and youth tobacco use are partially mediated by adversities (perceived discrimination, life trauma, financial strain). By exploring these relationships, we aim to provide insights that can inform policies and interventions aimed at reducing youth tobacco use and improving overall youth health.

## 3. Methods

### 3.1. Design and Sample

We conducted a secondary analysis using data from the Adolescent Brain Cognitive Development (ABCD) study, a national longitudinal study of a racially and economically diverse cohort of pre-adolescent children. The ABCD study's methodology has been thoroughly documented elsewhere. Advantages of the ABCD dataset include its longitudinal design, national scope, large and diverse samples in terms of race, SDOHs and geographic distribution. Participants were primarily recruited from schools. The analytical sample consisted of 6003 non-Latino White and 1562 non-Latino African American pre-adolescents. The study was approved by the Institutional Review Board (IRB) of the University of California, San Diego (UCSD). Assent was obtained from all participating adolescents, and informed consent was obtained from their parents.

### 3.2. Study Variables

The study variables included race, demographic and socioeconomic factors, adversities, and tobacco use. All ABCD participants were eligible for our analysis, regardless of race, ethnicity, or SDOHs (n = 11,878).

### 3.3. Predictors (Socioeconomic Status)

#### Family Structure:

Parents reported the number of parents in the household and their relationship. This was categorized as 0 for not married and 1 for married households.

#### Family Income:

Family income was measured on a 1-10 scale, where higher scores indicated higher income. The scale ranged from less than $5000 to $200,000+, as categorized by the ABCD study. This variable was treated as continuous.

#### Parental Educational Attainment:

Participants reported the highest level of education completed. Responses ranged from 0 (never attended) to 21 (doctoral degree), with higher scores indicating higher educational attainment.

#### Neighborhood Income:

Median family income in participants' zip codes was used, divided by 5000 for more interpretable beta coefficients.

Race Parents reported the race and ethnicity of their children. This was a categorical variable with White as the reference category.

### 3.4. Mediators (Stressors)

#### Life Stress.

Adverse life experiences were measured at baseline using a validated interview instrument that assessed traumatic events and adversities. This semi-structured interview included items such as family deaths, serious injuries, witnessing crimes, and losing close friends. Responses were coded as 0 (no) or 1 (yes), and follow-up questions determined the timing, positivity/negativity, and impact of these events. The total adverse life experiences score was calculated as a continuous measure, with higher scores indicating greater exposure to negative events.

#### Financial Stress.

Subjective family socioeconomic status (SES) was assessed through financial difficulties experienced in the past 12 months. Items included inability to afford food, telephone service, rent/mortgage, eviction, utility shutoffs, and unmet medical or dental needs. Responses were binary (0 = no, 1 = yes), and a mean score was calculated, with higher scores indicating greater financial stress.

#### Racial Stress.

Perceived discrimination was measured with seven items that assessed the frequency of unfair treatment due to ethnic background. Responses ranged from 1 (almost never) to 5 (very often), with a higher average score indicating greater perceived discrimination.

#### Perceived Neighborhood Stress.

Perceived neighborhood stress was assessed with three items, forming a continuous measure where higher scores indicated greater stress.

#### Family Stress.

Family conflict was measured using the Family Environment Scale, which included nine items assessing negative aspects of familial relationships such as fighting, anger, and criticism. Higher scores indicated greater family conflict. The Cronbach’s alpha for this measure was 0.681.

### 3.5. Outcome

#### Tobacco Use.

Tobacco use was measured semi-annually using instruments such as the iSay Sipping Inventory and a web-based Timeline Follow-Back (TLFB). These tools assessed substance use over the past six months or since the last study session. The analysis focused on tobacco use initiation, defined as reporting more than one puff of nicotine.

### 3.6. Data Analysis

Data analysis was conducted using SPSS. Univariate analysis involved reporting the mean and standard deviation (SD) of continuous measures. Structural equation models (SEM) were used for multivariable analysis, with tobacco use initiation as the outcome. Predictors included SES indicators (SDOHs), and mediators were various types of adversities. Age and gender were controlled for as confounders. Collinearity between variables was checked and ruled out (all correlations were below .6). Hazard Ratios (HR), 95% confidence intervals (CI), and p-values were reported.

## 4. Results

**Figure 1.**
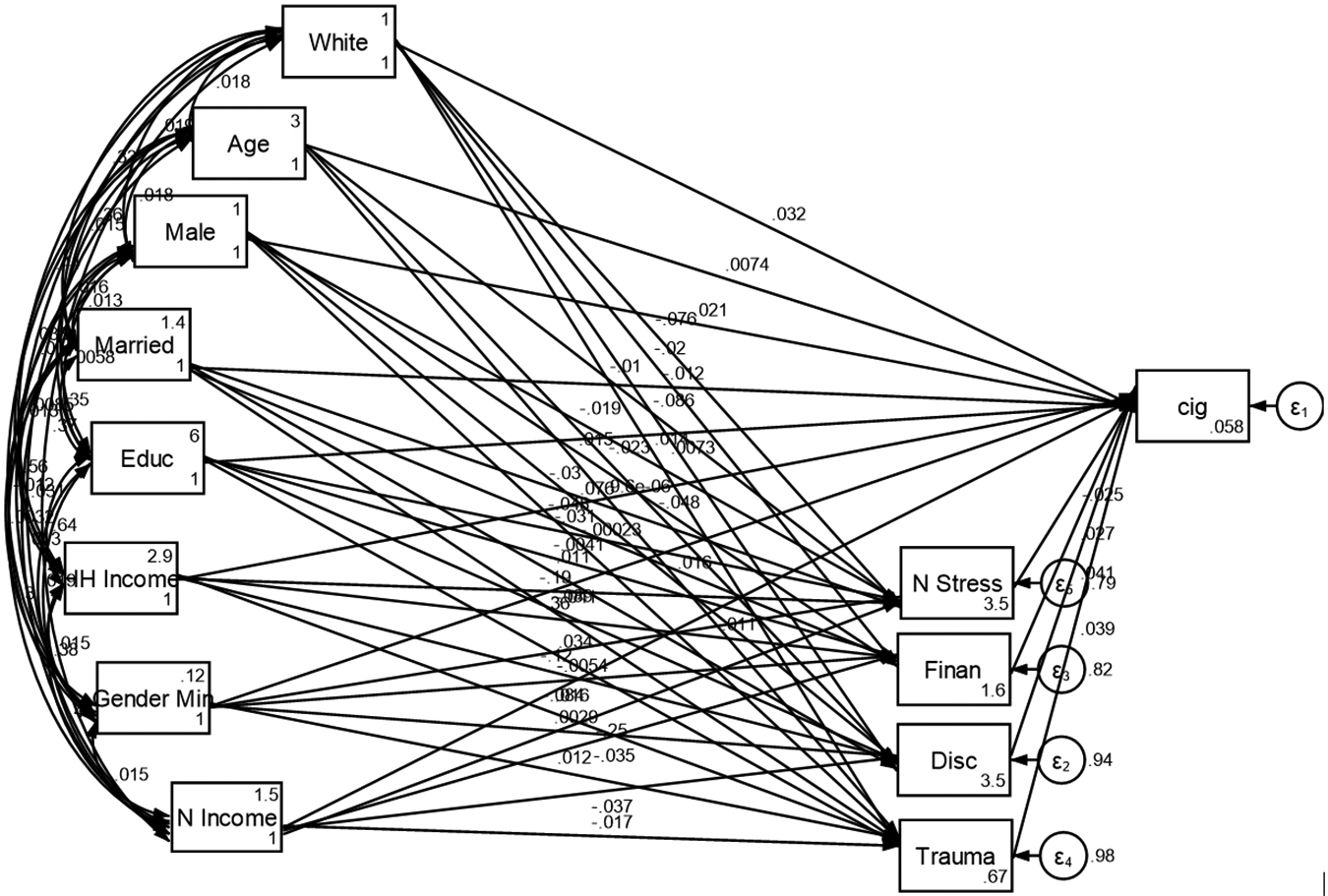
Adversities mediate the effects of SDoHs on tobacco use initiation

As shown by Figure 1, the effects of parental education, household income, neighborhood income, and family structure on youth tobacco use were partially mediated by adversities. Higher levels of parental education and household income were associated with lower tobacco use, and this relationship was weakened when accounting for adversities. Similarly, stable family structures and higher neighborhood income were linked to reduced tobacco use, with adversities playing a mediating role.

As shown by Table 1, the key adversities considered in the analysis include race/ethnic stress (discrimination), financial stress, life stress (trauma), and neighborhood stress. The effects of these adversities on tobacco use initiation were adjusted for several demographic and socioeconomic variables, such as zip code income, household income, race/ethnicity, age, sex, household marital status, gender minority status, and parental education at baseline.

Race/ethnic stress significantly predicted tobacco use initiation (B = 0.04, SE = 0.01, 95% CI: 0.02 to 0.06, p < 0.001), indicating that higher perceived discrimination is associated with an increased likelihood of initiating tobacco use. Financial stress also emerged as a significant predictor of tobacco use initiation (B = 0.03, SE = 0.01, 95% CI: 0.01 to 0.05, p = 0.010), suggesting that increased financial stress is linked to a greater risk of starting tobacco use. Similarly, life stress, measured by the number of traumatic events, was a significant predictor of tobacco use initiation (B = 0.04, SE = 0.01, 95% CI: 0.02 to 0.06, p < 0.001). Individuals experiencing more life stress were more likely to initiate tobacco use. In contrast, neighborhood stress had a negative association with tobacco use initiation (B = −0.03, SE = 0.01, 95% CI: −0.05 to 0.00, p = 0.015), indicating that lower neighborhood stress is linked to a lower likelihood of tobacco use initiation.

Higher household income was significantly associated with lower tobacco use initiation (B = −0.05, SE = 0.02, 95% CI: −0.08 to −0.02, p = 0.002). White participants were more likely to initiate tobacco use than their counterparts (B = 0.03, SE = 0.01, 95% CI: 0.01 to 0.05, p = 0.003). Male participants had a higher likelihood of initiating tobacco use compared to females (B = 0.02, SE = 0.01, 95% CI: 0.00 to 0.04, p = 0.020). Other factors, such as zip code income, age at baseline, being in a married household at baseline, gender minority status, and parental education at baseline, did not show significant effects on tobacco use initiation.

The analysis also examined how SES indicators such as income and education, relate to adversities like race/ethnic stress, financial stress, life stress, and neighborhood stress. Lower zip code income (B = −0.04, SE = 0.01, p = 0.001), lower household income (B = −0.12, SE = 0.02, p < 0.001), and lower parental education (B = −0.04, SE = 0.01, p = 0.001) were associated with higher race/ethnic stress. Both lower zip code income (B = −0.04, SE = 0.01, p < 0.001) and household income (B = −0.36, SE = 0.01, p < 0.001) were significant predictors of higher financial stress. Notably, married household status at baseline was associated with lower financial stress (B = −0.05, SE = 0.01, p < 0.001). Lower household income (B = −0.08, SE = 0.01, p < 0.001) and being in a non-married household at baseline (B = −0.09, SE = 0.01, p < 0.001) were associated with higher life stress. Lower zip code income (B = −0.25, SE = 0.01, p < 0.001), lower household income (B = −0.19, SE = 0.01, p < 0.001), and lower parental education (B = −0.03, SE = 0.01, p = 0.005) predicted higher neighborhood stress.

## 5. Discussion

The findings indicated that the effects of parental education, household income, neighborhood income, and family structure on youth tobacco use were partially mediated by adversities. Higher levels of parental education and household income were associated with lower tobacco use, and this relationship was weakened when accounting for adversities. Similarly, stable family structures and higher neighborhood income were linked to reduced tobacco use, with adversities playing a mediating role.

Our findings align with previous research highlighting the importance of social determinants of health in influencing youth tobacco use [15-19]. Parental education, household income, neighborhood socioeconomic status, and family structure significantly impact health behaviors and outcomes [19, 42-51]. These determinants shape the environment in which young people grow up, influencing their access to resources, exposure to stressors, and overall health trajectories [52-56]. Our study reaffirms the need to consider these broader social factors when addressing youth tobacco use.

The role of adversities, including perceived discrimination, life trauma, and financial strain, in youth tobacco use is evident in our findings. These stressors may contribute to psychological distress, which may lead to increased tobacco use as a coping mechanism [38]. Adolescents experiencing multiple adversities are at a heightened risk of initiating tobacco use, highlighting the need for targeted interventions that address these specific stressors [57-60].

The mediation analysis in our study indicates that adversities partially mediate the effects of SDOH on youth tobacco use. This suggests that the impact of parental education, household income, neighborhood socioeconomic status, and family structure on tobacco use operates through the pathway of adversities [54, 61-69]. Interventions aimed at reducing youth tobacco use should therefore consider strategies that mitigate the effects of these adversities, in addition to addressing the broader social determinants.

Our study supports the notion that tobacco use among youth can serve as a coping mechanism for stress. Adolescents facing high levels of perceived discrimination, life trauma, and financial strain may turn to tobacco use as a way to manage their stress and emotional distress [70-77]. Understanding tobacco use as a coping strategy underscores the importance of providing alternative coping mechanisms and mental health support to at-risk youth.

Stress is a multifaceted construct that spans various domains and has cumulative, additive effects on health behaviors [78-80]. Our study highlights the importance of considering multiple sources of stress, including perceived discrimination, life trauma, and financial strain, when examining youth tobacco use. These stressors do not operate in isolation but rather interact and compound each other’s effects, necessitating comprehensive approaches to stress reduction and support.

Policies aimed at improving the educational and economic situations of families, enhancing neighborhood environments, and supporting stable family structures play a crucial role in reducing youth tobacco use, primarily by lowering exposure to various adversities. Educational policies that increase access to quality education can provide youth with the knowledge and skills necessary to resist tobacco use and make healthier choices. Education also tends to improve future economic prospects, creating a pathway out of poverty and associated stressors. Economic policies that support stable and sufficient family income alleviate financial strain, a significant stressor linked to higher rates of tobacco use among youth. When families have adequate financial resources, they can better meet their basic needs, reduce stress, and provide a more stable home environment, all of which contribute to lower rates of tobacco use.

Enhancing neighborhood environments through policies that promote safe, supportive, and resource-rich communities can also have a profound impact. Neighborhoods with better access to recreational facilities, quality schools, healthcare, and social services provide youth with positive outlets and support systems, reducing the likelihood of turning to tobacco as a coping mechanism. Safe and well-maintained neighborhoods reduce exposure to violence and other traumatic experiences, which are known risk factors for tobacco use. Furthermore, community-based programs that foster social cohesion and collective efficacy can create a supportive environment that discourages tobacco use and encourages healthier behaviors.

Supporting stable family structures is another critical policy focus. Policies that promote family stability, such as those providing parental leave, affordable childcare, and family counseling services, help maintain a supportive and nurturing home environment. Stable family structures provide emotional and social support essential for youth development, reducing the need to seek solace in harmful behaviors like tobacco use. These policies also reduce the incidence of family disruptions and associated stress, further diminishing the risk of tobacco use.

The mediating role of reduced exposure to adversities explains the effectiveness of these policies in lowering youth tobacco use. Adversities such as perceived discrimination, life trauma, and financial strain are significant stressors that increase the likelihood of tobacco use as a coping mechanism. By addressing the root causes of these adversities through educational, economic, and community-based policies, we can mitigate their impact on youth. For instance, reducing financial strain through economic policies lessens the chronic stress that drives youth towards tobacco use. Similarly, improving educational opportunities and neighborhood environments reduces the exposure to life trauma and perceived discrimination, fostering a sense of security and well-being that is incompatible with the need to use tobacco as a coping strategy.

### 5.1. Implications

Enhancing the educational and economic conditions of families, improving neighborhood safety, and support stable family structures are effective in reducing youth tobacco use. These policies work by addressing and reducing the adversities that contribute to tobacco use, providing a multifaceted approach to improving youth health and well-being. By creating supportive environments at home, in school, and in the community, these policies lay the groundwork for healthier future generations, demonstrating the interconnected nature of social determinants of health and behavioral outcomes.

The findings from our study have important policy and clinical implications. Policies aimed at reducing youth tobacco use should address both the social determinants of health and the adversities that mediate their effects. This includes efforts to improve educational and economic opportunities, enhance neighborhood environments, and support stable family structures. Clinically, providing mental health support and stress management resources to adolescents can help mitigate the impact of adversities and reduce tobacco use as a coping strategy.

### 5.2. Limitations

While our study provides valuable insights, it has several limitations. The cross-sectional nature of the data limits our ability to infer causality. Additionally, self-reported measures of tobacco use and adversities may be subject to bias. Future research should employ longitudinal designs and objective measures to validate our findings. Moreover, the generalizability of our results may be limited to similar populations and settings.

## 6. Conclusions

In conclusion, our study demonstrates that adversities partially mediate the effects of social determinants of health on youth tobacco use. Addressing both the broader social determinants and the specific adversities faced by adolescents is crucial for developing effective interventions to reduce tobacco use and improve youth health outcomes. By understanding and targeting these complex pathways, we can better support the well-being of young people and promote healthier behaviors.

## Figures and Tables

**Table 1.** Adversities mediate the effects of SDoHs on tobacco use initiation

		B	SE	95%	CI	P
Tobacco Use (Over Time)						
	Race/Ethnic Stress (Discrimination)	0.04	0.01	0.02	0.06	< 0.001
	Financial Stress	0.03	0.01	0.01	0.05	0.010
	Life Stress (Trauma, n)	0.04	0.01	0.02	0.06	< 0.001
	Neighborhood Stress	−0.03	0.01	−0.05	0.00	0.015
	Zip Code Income / 50000	−0.01	0.01	−0.03	0.01	0.331
	Household Income	−0.05	0.02	−0.08	−0.02	0.002
	Race/Ethnicity (White)	0.03	0.01	0.01	0.05	0.003
	Age (10 Yrs) at Baseline	0.01	0.01	−0.01	0.03	0.419
	Sex (Male)	0.02	0.01	0.00	0.04	0.020
	Married Household at Baseline	−0.01	0.01	−0.03	0.01	0.266
	Gender Minority	0.02	0.01	0.00	0.03	0.072
	Parental Education at Baseline	0.01	0.01	−0.02	0.03	0.549
	Intercept	0.06	0.08	−0.10	0.22	0.478
						
Race/Ethnic Stress (Discrimination)						
	Zip Code Income / 50000	−0.04	0.01	−0.06	−0.02	0.001
	Household Income	−0.12	0.02	−0.15	−0.09	< 0.001
	Race/Ethnicity (White)	−0.09	0.01	−0.11	−0.07	< 0.001
	Age (10 Yrs) at Baseline	−0.02	0.01	−0.04	0.00	0.014
	Sex (Male)	0.08	0.01	0.06	0.09	< 0.001
	Married Household at Baseline	−0.01	0.01	−0.03	0.01	0.350
	Gender Minority	0.00	0.01	−0.02	0.02	0.755
	Parental Education at Baseline	−0.04	0.01	−0.06	−0.02	0.001
	Intercept	3.48	0.07	3.35	3.61	< 0.001
						
Financial Stress (n)						
	Zip Code Income / 50000	−0.04	0.01	−0.05	−0.02	< 0.001
	Household Income	−0.36	0.01	−0.39	−0.34	< 0.001
	Race/Ethnicity (White)	−0.02	0.01	−0.04	0.00	0.043
	Sex (Male)	0.02	0.01	0.00	0.03	0.072
	Married Household at Baseline	−0.05	0.01	−0.07	−0.03	< 0.001
	Gender Minority	0.02	0.01	0.00	0.03	0.049
	Parental Education at Baseline	0.00	0.01	−0.03	0.02	0.712
	Intercept	1.63	0.05	1.53	1.73	< 0.001
						
Life Stress (Trauma, n)						
	Zip Code Income / 50000	−0.02	0.01	−0.04	0.00	0.112
	Household Income	−0.08	0.01	−0.11	−0.06	< 0.001
	Race/Ethnicity (White)	0.01	0.01	−0.01	0.03	0.191
	Age (10 Yrs) at Baseline	0.00	0.01	−0.02	0.02	0.999
	Sex (Male)	0.00	0.01	−0.02	0.02	0.980
	Married Household at Baseline	−0.09	0.01	−0.11	−0.07	< 0.001
	Gender Minority	0.01	0.01	−0.01	0.03	0.202
	Parental Education at Baseline	0.03	0.01	0.01	0.06	0.005
	Intercept	0.67	0.06	0.54	0.80	< 0.001
						
Neighborhood Stress						
	Zip Code Income / 50000	−0.25	0.01	−0.27	−0.23	< 0.001
	Household Income	−0.19	0.01	−0.22	−0.17	< 0.001
	Race/Ethnicity (White)	−0.08	0.01	−0.09	−0.06	< 0.001
	Age (10 Yrs) at Baseline	−0.01	0.01	−0.03	0.01	0.212
	Sex (Male)	−0.02	0.01	−0.04	0.00	0.019
	Married Household at Baseline	−0.03	0.01	−0.05	−0.01	0.002
	Gender Minority	−0.01	0.01	−0.02	0.01	0.511
	Parental Education at Baseline	−0.03	0.01	−0.05	−0.01	0.005
	Intercept	3.46	0.06	3.35	3.57	< 0.001
